# Relationships between Depression and High Intellectual Potential

**DOI:** 10.1155/2012/567376

**Published:** 2012-05-08

**Authors:** Catherine Weismann-Arcache, Sylvie Tordjman

**Affiliations:** ^1^Clinical Psychology “Individual and Family Trauma” Team, Laboratoire Psychologie et Neurosciences de la Cognition et de l'affectivité (EA 4306), Rouen University, 76821 Mont Saint Aignan, France; ^2^Clinical Psychology and Psychopathopsychology Laboratory, Paris Descartes University, 75015 Paris, France; ^3^Department of Child and Adolescent Psychiatry, Centre Hospitalier Guillaume Regnier and University of Rennes 1, Rennes, France; ^4^Laboratoire de Psychologie de la Perception, CNRS UMR 8158, Paris Descartes University, Paris, France

## Abstract

This paper proposes to analyse the relationships between depression and high intellectual potential through a multidisciplinary and original approach. Based on their respective experience in psychology and child psychiatry, the authors will focus their analysis on creative potential. First, relationships between creativity (literary, artistic, or scientific creativity) and melancholy (“melancholy” comes from the Greek words for “black” (“melas”) and “bile” (“khole”)) will be examined from antiquity to modern times. Aristotle introduced a quantitative factor, asserting that levels of melancholy and black bile are positively correlated; however, under a given threshold of black bile, it can give rise to an exceptional being. Second, the case study of Blaise Pascal (scientific and philosophical creativity associated with major depressive episodes from childhood) will be presented and discussed. This case study sheds light on the paradoxical role of depression in the overinvestment in intellectual and creative spheres as well as on the impact of traumatic events on high intellectual potential. Third, observations will be reported based on a study conducted on 100 children with high intellectual potential (6–12 years old). Finally, based on these different levels of analysis, it appears that heterogeneity of mental functioning in children with high intellectual potential is at the center of the creative process and it has related psychological vulnerability.

## 1. Introduction

Childhood depression is still a relatively taboo subject in clinical practice, its existence long denied by parents and professionals alike. High intellectual potential is another, idealizing measure leading to both resistance and fascination. Whereas childhood depression is now recognized as a distinct pathopsychological entity, its descriptions are many and various, changing with each new classification and epistemology. As for high intellectual potential, although a consensus has been reached over its psychometric definition—an intelligence quotient (IQ) above 130, according to the criterion of the World Health Organization (WHO)—different conceptions continue to exist, depending on the intellectual, developmental, cognitive, factorial, or dimensional model to which one refers. There is a very wide gap between authors who adopt a descriptive and symptomatic approach to these two “dimensions” and those who seek instead to understand their underlying psychological processes from a holistic perspective. Diagnoses of depression and/or high intellectual potential may thus be clinical, psychiatric, or psychodynamic. It should be noted that the terms “depression” and “high intellectual potential” are not found in the vocabulary of psychoanalysts, who prefer to talk about melancholy and loss on the one hand, and thought-cathexis, epistemophilic impulse, or sublimation on the other. Whereas we seek to define these various conceptions in the present paper, above all we discuss the implicit link that is frequently drawn between depression/anxiety and high intellectual potential, not just in the minds of lay people and the media, but in the specialist literature, too. Paradoxically, studies of high potential often focus on young people attending special clinics and who are therefore subject to psychological problems, even though a large proportion of such children seemingly experience no particular difficulties. This introduces a definite research bias and means that there is little empirical research conducted on the latter population [1]. For this reason, in our own research on high potential, we have been comparing a group of children referred to the National Center for children with high intellectual potential (CNAHP; coordinated by S. Tordjman) for problems at school and psychoaffective (depression and/or anxiety disorders) and behavioral (hyperactivity and/or aggressive conduct) difficulties, with a control group who originally came to a private practice for a psychological assessment (carried out by C. Weismann-Arcache). Each group currently comprises around 50 children (i.e., approximately 100 participants in all), aged 6–12 years. It should be noted that just because a child has never been referred to a specialist does not mean that he or she has no psychological problems. This article is based on the general data we have collected from our samples so far and on what we know about Blaise Pascal. This author, Blaise Pascal, a “terrifying genius” for Chateaubriand and a “sublime misanthrope” for Voltaire, exhibited major depressive symptoms from early childhood (the misanthrope), associated with high scientific and philosophical potential (the genius).

We explore the implicit link drawn between depression and high potential both longitudinally (from antiquity to the present day) and transversally looking at the various parallels and interactions that can be established between these two entities. We examine causality in a nonlinear manner, deeming that high potential and depression, far from being “superstructures” encompassing the whole of an individual's psychological organization, are actually aspects of the developing personality that are modified and colored in a nonstandard way according to that person's individual characteristics. We cannot regard depression as a manifestation of high potential without immediately evoking the opposite hypothesis whereby high potential is a (defensive?) manifestation of depression. According to Tordjman [2] high potential may be either the cause or the consequence of psychopathological disorders. In the case of children with high potential with problems, two questions spring to mind:

“*Can atypical and precocious cognitive development disturb affective development? Or is it the other way round, with disturbed socio-affective development resulting in cognitive overinvestment? It may well be that early social interaction deficits lead children to become socially isolated which, in turn, triggers intellectual hypercathexis. Conversely, because of their differencenot least their “gifted” status and identity, possibly maintained by their immediate circle—precocious children may be rejected by their social environment and find themselves isolated. This may lead to the feelings of persecution that are exhibited by many children with high potential in situations of exclusion or victimization. It is difficult to disentangle the respective contributions of cognitive and affective factors to the development and expression of high intellectual potential, not least because these two aspects may start to feed into each other over time, creating a veritable vicious circle.*” [3, page 12].

We therefore highlight the reciprocal incidences of high potential and depression, describing the characteristics of the underlying defensive organization in accordance with the psychodynamic model of child development: impulse—reaction formation (defense mechanisms)—character formation sublimation. Above and beyond the extreme or overdeveloped nature of certain aptitudes and certain components of these children's psychological organization, we underscore the heterogeneity of their mental functioning, in terms of excess and deficit, pressure and depression.

## 2. Genius and Melancholy

A link between depressive disorders and literary, artistic, or scientific genius has been drawn since Antiquity. Hippocrates' “black bile” in the 4th century B.C., the “acedia” of the Middle Ages and the Renaissance, and the “spleen” or “*mal du siècle*” of the 19th century Romantics were all variations on a theme of despondency giving rise to dark and sorrowful thoughts. Since Antiquity, the accepted term for this condition has been “melancholy”, a term made up of the Greek words for “black” (“*melas*”) and “bile” (“*khol*ē**”) [4]. From the very outset, melancholy was assumed to draw its sustenance from the finitude and distress inherent to the human condition, and fuel thinking and philosophizing on the existential enigmas of humanity. In more recent times, Freud and his successors showed that thinking and fantasizing stem from real and imagined losses. When we move from one developmental stage to the next, we have to relinquish the previous mode of satisfaction, in what is an entirely normal depressogenic process. This means that certain periods in normal child development, such as early childhood and adolescence, are characterized by particularly acute vulnerability. As a result, psychopathological studies of depression have tended to focus on infants and adolescents, to the detriment of children. It is worth noting that early childhood and adolescence are also periods of intense and rapid cognitive transition, when mental functioning is characterized by considerable hetereogeneity, or “unleveling”, to quote Anzieu [5] who observed the same phenomenon in creative individuals. We believe that this heterogeneity, which we have also identified in children with high potential, lies at the heart of the creative process and its relased psychological vulnerability. We reject the hypothesis that such children suffer from affective immaturity, pointing instead to tensions within their mental apparatus:

“*The dyssynchrony said to characterize the conduct of children with high potential is, in fact, extremely polymorphous. While it sometimes signals a cognitive dysfunction, more often than not, it masks a heterogeneity inherent to the development of high-level intelligence. The heterogeneity of mental functioning in children with high potential is thus akin to the teenage crisis or to the un-leveling observed in creative individuals*” [6, page 489].

The commentaries on the Aristotle's text on melancholy and genius [7] asks “why is it that all those who have become eminent in philosophy or politics or poetry or the arts are clearly of an atrabilious temperament?,” likening the effects of “black bile” to those of wine, insofar as it can bring to light a person's latent dispositions. In his new translation and commentary, Aristotle [7] eschews the expression “juice of the grape” in favor of “humor of the grape”a pun referring not just to darkness but also to the drunkenness that characterizes the mania said to arise from an excess of hot yellow bile, bearing in mind that the latter can temper cold black bile to make the explosive mixture found in exceptional individuals. Aristotle discusses two points which seem just as relevant as ever, namely, the particularly reactive constitution of exceptional individuals and the manner in which their basic receptivity is triggered by external and internal stimuli.

He begins by emphasizing the existence of a basic “state” that is activated by events. Thus, wine may call forth a wide variety of moods, ranging from mania (hyperthymia) to despondency (dysthymia), “making men, for instance, irritable, benevolent, compassionate or reckless.” This is the arousal of a potential for trauma that can be observed in children of high potential. Aristotle then introduces a quantitative factor, asserting that the more concentrated the “admixture” of black bile, the more melancholic a person will be. Providing it does not exceed a given concentration, however, this black bile will give rise to an exceptional being. Way ahead of his time, Aristotle introduces the distinction between state and disposition that is now part of our “high potential” terminology, with its assumption of unactualized dispositions, and concludes that this natural and lasting state, wherein melancholy is associated with exception, is one of paradoxical constancy.

He goes on to highlight a further paradox, in that this constancy holds within it an inconstancy, for this potential partakes of a “natural” state that makes it easier for individuals to identify themselves with other people. The gifted melancholic thus has a propensity to become “the others, all the others.” Aristotle [7] associates this imitation with the ability to construct representations of the world, the aim being to model oneself in order to become somebody different and to recreate the world through representation. Freud said much the same thing when he likened the child at play to “a creative writer, in that he creates a world of his own, or, rather, re-arranges the things of his world in a new way which pleases him” [8, page 34]. Alongside this lability of identification, Aristotle appears to describe a sort of intellectual hyperactivity, insofar as genius and melancholy become exponential. According to Aristotle [7], the melancholic engages in a desperate race to flee the “bite of the black bile,” requiring ever higher doses of entertainment, violence and contrast. The extreme and often disharmonious conduct of children with high potential, and the way in which they seek refuge from depression in intellectualization or sublimation, is all too clearly foregrounded in this seminal text.

## 3. Blaise Pascal and Depression in Early Childhood

Current theories of trauma stemming from excess or deficitfactors which some authors, such as Jean Bergeret [9], have linked to depression in so-called borderline personality disorder can be traced back to the ideas developed by Aristotle and discussed at the start of this paper. Furthermore, in a successful bid to reconcile the paradoxes and even quarrels arising from this concept, Janin [10] has used the Greek philosopher's hot-and-cold metaphor to devise the concepts of “hot nucleus” and “cold nucleus.” According to Janin, the cold nucleus of trauma forms as a result of an early inadequate relationship, which opens the floodgates to an unmanageable excitation that subsequently constitutes the hot nucleus.

“*According to my conception, the first stage in the trauma is characterized by a failure to respect these patients' needs as children and results in narcissistic injury: this is the cold nucleus of the trauma that has not been assimilated by the ego. The second stage consists of the sexualization of the first traumatic stage, and may take different forms in different patients […, this I have named the hot nucleus.” *Claude Janin continues, *“The third stage of the trauma takes place during puberty and consists of the constitution of a paradoxical trauma made up of these two hot and cold nuclei, which then become indistinguishable.” *

Aristotle's quantitative and medical model has thus become an economic model, in which his “basic state” can be equated with the constitutional strength of the drive. This excitability potentially opens up breaches that can become traumatic places, but which are necessary if individuals are to be even minimally receptive to relationships, to the world and indeed to their own selves. Whether it stems from lack or from excess, whether it is internal or external, narcissistic or sexual, trauma faces, both the past with the lost object and also the future with the realization of desiresits “afterwardsness” (theory of deferred action). These two perspectives are not as diametrically opposed as one might think and apply not just to trauma, but to the whole of clinical psycho-pathopsychology, as well as to the various depressive disorders. For affects, too, have a history, with anxiety anticipating a disquieting future and depression arising from the loss of a previous satisfaction. Still on the subject of history, Golse [11] argues that since the fifties, we have witnessed a shift from an orificial psychoanalysis, interested in orifices (i.e., erogenous zones of each partial drive), where neurosis and content were the clinical features, to a more skin-deep, Winnicottian psychoanalysis, focusing on containers and envelopes, and therefore on “hollow” traumas, with the holes and porosity of the skin ego. However, there cannot be hollows without bumps, and Cournut [12] contends that emptiness, and a dead, mourning, absent or depressed mother, may simply be the hidden face of excess, and an overspilling of yearning (Freud's “*Wunsch*”). “Burning sister to an icy mother” is what he writes on the subject of the Wolf Man, underlining the frequency with which seductive sisters make up for austere mothers.

This brings us to Blaise Pascal, as we know that he enjoyed a passionate and possibly even incestuous relationship with his sister, after losing his mother at an early age. Blaise's childhood prefigured a destiny of excess and lack. He was a puny, delicate infant in whom the notion of “helplessness” took its full traumatic meaning, as he fell into “mesenteric disease” at the age of one year. Anzieu [5] linked this “decline” to anaclitic depression, a concept first developed by René Spitz (1968). Pascal's biographers report that he was saved from the cold hand of death by a scalding poultice applied by a bonesetter. Here, then, was a child who was exposed to the contrasts of hot and cold, and their “Aristotelian” consequences, from a very early age. Subsequently he developed two phobias, one concerning his parents' expressions of love for each other (excitation excess), the other running water, evocative not just of infantile sexual theories, but also of the fear of “emptying out” (emptiness). Pascal's scientific and philosophical thinking was antitraumatic, in that he worked on the very subjects that caused him anxiety and uncertainty, namely, emptiness, probability, and the transformation of liquids, solids, and gases which, according to Anzieu [5], correspond to the urine, flatulence, and excrement that flow uncontrolled and uncontrollable from the body of a tiny child. We can speculate that the young Blaise had a constitutional hyperreactivity that rendered him particularly receptive, with the result that normal situations, such as the loving relationship between his parents, became wounding ones to him. If that was the case, we can easily imagine the impact of his mother's death when he was just three years old. As Bailly [13] has written:

“*The baby is in his cradle, expecting his mother to come whenever he wishes. He babbles, but she does not come. He cries, but she does not come. He screams, but she does not come. She no longer comes. She will never come again*.”

According to Bailly this type of traumatic loss questions sometimes even damages infantile sexual theories. However, these theories can also be intensified by the omnipotence of thought. From a very early age, Pascal displayed true scientific and philosophical genius. A precocious mathematician, he developed the theory of probability, which he later applied to his metaphysical concerns about the existence of God, in his famous Wager. It should be pointed out that children with high potential perform particularly well on the Piagetian-type “Probability Quantification” cognitive tests, on account of their tremendous need for certainty and anticipation [14]. Pascal the physicist tackled the problem of loss by seeking to demonstrate the existence of vacuum, and Pascal the theologian by attempting to prove the existence of God, possibly as an antidote to that vacuum. He wanted to test—and, if possible, invalidate—Aristotle's precept that “nature abhors a vacuum.” We could compare these two geniuses, for while one worked on the different permutations of the humors, and the other worked on gases, liquids, and solids, which, according to Anzieu [5], also referred to the bodily humors, early bodily fears of emptiness and repletion, and the quest for a center of gravity, in Pascal's case. We believe that the origins of his overdeveloped scientific and metaphysical thinking lay in the depression he suffered in early childhood and which continued to gnaw away at him throughout his short life, mobilizing extreme narcissistic defenses to the point of no return (he attacked his own body). It was the positive use of depression that led him to pen his *Pensées* [15].

According to Ferenczi [16, 17], children develop wisdom beyond their years in response to adults' inability to satisfy their needs. This may involve not only an absence of love, but also inadequate, excessive, or abusive forms of adult love for a child. Freud's colleague had come up with his theory as a result of his clinical experience with patients who had been physically or sexually traumatized and extended this notion of abuse to cover other distortions in the parent-child relationship. He described the so-called “wise baby” [16], which develops a split-off intellectual precocity that serves to protect it from the adults' madness and incoherence.

“*On the emotional but also the intellectual level, the shock may allow part of the individual suddenly to mature. I wish to remind you of the typical ‘dream of the wise baby' described by me several years ago, where a newborn, a child still in the cradle, suddenly starts to talk and in fact teaches wisdom to the entire family. The fear of the uninhibited, almost mad adults, changes the child, so to speak, into a psychiatrist. To protect himself from the danger represented by the uninhibited adults, he must know how to identify himself completely with them*.”

The dangers of this lability of identification, which Aristotle evoked in rather different terms, are all to easy to see in high potential children with problems, and include the risk of overadapting to others, if not adopting a “false self” that stifles one's desires and genuine personality, and the risk of becoming diluted in these multiple identifications and experiencing feelings of emptiness, lack of consistency, and even depersonalization. The mortification that the adult Blaise Pascal inflicted upon himself was doubtless a way of recentering himself and containing his anxieties [18].

## 4. Clinical Features of Children with High Potential: Pressure and Depression

As we do not see high intellectual potential as a distinct nosographic entity, we selected our participants on the basis of a single criterion and did not prejudge the homogeneity of our sample. This criterion was psychometric and quantitative, namely an IQ above 130, even though researchers have started to make a distinction between academic and nonacademic (e.g., creative) high potential [2, 19]. Every child initially underwent an in-depth psychological investigation designed to objectivize high intellectual (and creative) potential and assess overall mental functioning, by means of clinical interviews, the Wechsler Intelligence Scale for Children (WISC), Piagetian tests, projective tests (Children's Apperception Test (CAT) or Thematic Apperception Test (TAT) and Rorschach, drawings (child's family, Rey-Osterrieth Complex Figure Test, Dame de Fay), and a psycho-affective assessment (depression, self-esteem, and anxiety questionnaires). In addition, a direct observation of the child completed by a parental interview was conducted by two child psychiatrists. The presence of major depressive disorder according to DSM-IV-TR criteria was found in 65% of the 100 children with high intellectual potential. It is noteworthy that this high frequency of major depressive disorder was observed in the children with high intellectual potential who came to the CNAHP for psychoaffective problems, but also in the children with high intellectual potential who came to private practice for psychological assessments to skip a grade. This finding underlines the importance to study the relationships between depression and high intellectual potential. However, this high frequency of major depressive disorder concerns only children with high potential who came for professional advice, and therefore this result should not be extended to all the children with high potential.

By focusing on “anachronisms” as the vectors of pathology and sublimation, we have so far been able to identify several shared characteristics, with a variability ranging from the normal to the pathological [20]:

- intense questioning about origins and finitude versus emptiness, lack, and castration, which continues to nourish relatively unrepressed infantile sexual theories for a considerable length of time, and a particular relationship with time, colored by existential anxiety about separation, loss, and death;
- a counterphobic use of thought and language in an attempt to shore up a libidinal impulse in search of self-soothing or a protective shield to contain fears of loss. Object relations are traumatic in nature, characterized by excess/lack in a way that is reminiscent of the game of hide and seek in which the child burns if he or she gets close to the desired object and freezes if he or she moves away from it;
- avoidance/refusal of models in all fields, in an illusory attempt to elude dependency on reality and objects. These children favor their own mental representations, which not only form the bedrock of their identity and identification, with all its attendant vagaries, but also constitute one of the conditions for creativity, if not genius. At the clinical level, the rejection of models may manifest itself in poor writing and copying skills, noncompliance with school rules, and a reluctance to draw or to play symbolic games. Trial and error is not permitted, as it represents the gap between the model to be attained—the ideal—and the limitations of the child's ego.

Ours is a heterogeneous population covering the whole range of psychopathological classifications, from variations of normality to the most severe disorders. This same transnosographic “cross-border” dimension of high potential can be found in siblings containing several individuals with overdeveloped intelligence, as the latter may present with a variety of disorders, including what are currently labeled dyspraxia, ADHD, and high-functioning autism. Our experience shows also that most of the requests for consultations we receive from families with high potential children concern separation difficulties, ranging from very slight to extremely severe. The symptoms presented are recurrent “boredom,” “loneliness,” “lack of self-confidence” and “sleep disturbances.” Separation difficulties, notably lack of self-confidence, generally manifest themselves at nursery school entry, when forced socialization and classroom learning bring to light a lack of wellbeing which the child's family had either not picked up on or had assumed to be a token of eccentricity or exception, as high potential can easily mask underlying depressive features with its precocious logic and rationalization [2].

The same is true, albeit to a lesser extent, for “nongifted” children with depression, for the younger the child, the more the symptoms express his or her struggle against depression. In line with Aristotle's observation that “the young are more cheerful, the old more despondent, the former being hot and the latter cold,” the symptoms they display are agitation, aggression, and oppositionalitythe very opposite of downheartedness and neurasthenia. Sets of depressive symptoms may also vary according to the children's personality development and organization, taking the form of action and oppositionality, withdrawal and inhibition, or somatic disorders and school, social, or familial maladjustment. These categories can be divided further into depressive behavior, antidepressive behavior, depressive equivalents, masked depressions, and so on, regardless of whether the depression is neurotic, psychotic, or borderline, or takes the form of a major depressive episode. One of the hotly debated topics at the present time concerns the nature of depressive affect. Should it be viewed as a basic affect, in line with John Bowlby's attachment model or, as Denis claims [21], as working-over, based more on a model linked to relational development? Whatever the case, given that depressive symptomatology changes with age, it is important to distinguish between depression in infants, children and adolescents.

Depression is just as difficult to diagnose in adolescents as it is in individuals of high potential, as depressed mood is “normal" in this age group and brings with it a risk of transition to more typical disorders. To complicate matters further, we have shown that high intellectual potential is accompanied by precocious psychosexual development, with children displaying the affective characteristics of adolescents [14, 15]. In every case, the diagnosis of depressive disorders requires a two-pronged approach.

1. A descriptive and symptomatic approach, identifying those symptoms that are suggestive of a “depressive state” (i.e., a pathopsychological disturbance), bearing in mind that children's depressive symptomatology is both broad and varied. In a previous study [22], we examined a sample of young children of high potential consulting for adjustment problems at nursery school who simultaneously met the French classification of Child and Adolescent Mental Disorders (CFTMEA-R 2000; Misès et al., 2002) criteria for separation anxiety and the DSM-IV-TR (American Psychiatric Association, 2004) criteria for major depressive disorder:
  - CFTMEA-R 2000 Axis I (7.4) *behavior and conduct disorders; separation anxiety disorders*: prolonged and intense manifestations of anxiety, such as crying, boredom, disturbed social functioning, and sleep disturbances;
  - DSM-IV-TR: loss of pleasure, feelings of worthlessness, inability to concentrate and thoughts of death.
2. A clinical pathopsychological approach, whereby an explanation for this “depressive episode” is sought in the individual's history, without neglecting his or her environment. This investigation may bring depressive aspects to light that were hitherto masked by what can be extremely polymorphous symptoms. Adopting a psychoanalytic angle, an attempt is made to link the depressive disorder to a neurotic, borderline, or psychotic organization. Bergeret's model [9, 23] provides for just such a classification (neurotic depression triggered by castration anxiety, borderline depression triggered by loss anxiety, and psychotic depression triggered by identity anxiety). Depression and borderline states actually tend to be subsumed under the heading of “narcissistic pathologies” in this model, but other authors do make a distinction between narcissistic pathologies and borderline ones, asserting that they have different defensive structures, especially concerning dependency on others and separation. Fear of loss is the common denominator, and it is the way in which loss itself is dealt with that distinguishes the different clinical forms of depression apart [24]. For, as Jacques André stresses [25], depression is the product not so much of loss as of the impossibility of integrating that loss. The same is true for separation.

In children with high potential, fear of loss emerges at an early age. Both intense and longlasting, it is accompanied by an acute awareness of the passage of time. Children with high potential are nostalgic creatures, extremely conscious of the fact that each present moment slips through their fingers before they have had a chance to experience it. “The next second is already the future” exclaimed one six-year-old boy, and there are all those who want to be paleontologists, geologists or archeologists when they grow up, or who want to invent time machines or antipollution devices that will restore Earth to its primal, pristine condition. This “search for lost time” is also reflected in an interest in planets, prehistory, mythology, and other topics relating to the mysteries of appearance and disappearance. This nostalgia can lead to genuine bouts of depression, as in the nine-year-old boy of high potential who experienced crying spells and even suicidal thoughts at the end of every weekend, especially if there had been a large family gathering or celebration. Anxiety about growing up, growing old, and dying is frequent, and children sometimes see their worst fears confirmed, with the death of a loved one. This is certainly what happened to Blaise Pascal, and the biographies of other men and women famed for their intellectual, artistic or scientific prowess often make mention of the early loss of a beloved mother, father or sibling—a phenomenon that Brenot [26] has dubbed “orphaned genius.” These traumatic losses appear to trigger fruitful inner work, mobilizing creativity, and the omnipotence of thoughtsa sort of mental survival kit. Another child with high potential had taught himself to read at the age of three by deciphering his grandmother's daily newspaper. She had been looking after him while his father lay in hospital, close to death. This child had therefore learned to read in extreme circumstances, impelled by an imperious and vital need, possibly associating adult knowledge with an omnipotence that would avert the worst-case scenario. Many artists and writers turn out to have been orphans. Tolstoy, for instance, was left motherless at an early age, as were Nerval, Michelangelo, and Stendhal. Similarly, Sartre, Newton, Camus, Mauriac, Nietzsche, and Sand all lost their fathers in their early childhood or even before they were born. Not all orphans go on to be children or adults with high potential, whereas not all individuals with high potential have experienced bereavement. Nonetheless, sensitivity to loss and thence to relationships is a constant which may lend a traumatic coloring to grief and happiness alike.

Loss may also occur within relationships, in the shape of irregular and even chaotic psychological availability. In the course of the consultations we provide for children and adolescents with high potential, we have found that maternal depression and a family history of bipolar disorders are relatively frequent, and Tordjman [19] has underscored the interdependence of creativity and vulnerability to bipolar disorders involving manic episodes associated with major depressive episodes. André Green's “dead mother complex” [27] is predicated on a brutal loss of attention and maternal love within a hitherto fulfilling relationship. In the wake of this loss, the infant constructs a defensive and protective structure (“patched breast”) founded on a “compulsion to imagine” and/or “compulsion to think.” To illustrate this flow of “pressure/depression,” we have devised a triptych entitled “wooed child, phobic child, gifted child” [28] suggesting a possible link between hypersensitivity to relationships and defensive, counterphobic cathexis of thought. Geniuses in different fields have often been “wooed” as children, whether this seduction is real and traumatic, as in the case of *Œ*dipus, who solved the Sphinx's riddles so intelligently, or initiatory, as in the case of Leonardo da Vinci, who is said to have received many passionate kisses from his mother, or Freud, the beloved son of Amalia, whose own experience prompted him to write that [29]:

“*If a man has been his mother's undisputed darling, he retains throughout life the triumphant feeling, the confidence in success, which not seldom brings actual success along with it.*”

For Anzieu and Besdine [30] the mothers of creative individuals are “potential Jocastas,” whose maternal love acts as a powerful stimulant. Children with high potential have often been long awaited, and many of our participants were born as a result of assisted reproductive technology, leading us to come up with the adage “late motherhood, precocious children!” This intensive mothering may continue way beyond early childhood, in an almost incestuous mode. It is equally plausible that mothers may unconsciously respond to particularly high receptivity in their infants—a virtually constitutional receptivity which may be present from the very outset. The happiness of their early years can make children with high potential extremely nostalgic and generate feelings of guilt, in that sons and daughters will often have formed couples with their mothers. This apparent merger may conceal the impossibility of foregoing the narcissistic perfection of childhood and of renouncing the ideal child for the mother, which weighs heavily on the child's self-representation. A narcissistic form of depression may thus emerge from the gulf between the child's actual ego and the inaccessible ego ideal. So-called narcissistic defenses are then erected, with high potential individuals frequently having recourse to idealization, denial of lack and suffering, refusal of completeness and narcissistic “splendid isolation.” Early and massive cathexis of symbols would thus appear to signal a quest for independence and for control over the external object—the very object that may become wanting, leaving the child prey to an excess of yearning (“*Wunsch*”; Freud, 1926).

## 5. Conclusion

In conclusion, the depression observed in children with high potential would seem to be characterized by narcissistic vulnerability associated with genuine traumatophilia, which Lowenfeld [31] defined as “a degree of traumatic susceptibility exceeding the normal.” This author based this notion on the fact that some artists repeatedly confront trauma in order to overcome it through elaboration and symbolization. The same notion has been put forward by Michèle Emmanuelli. In her research on thought processes in adolescence [32], she compared traumatophiliac tendencies in artists and adolescents, looking at how these promote sublimation and creativity. We can hypothesize that an artist seeking to alleviate the trauma of loss in early childhood has no choice but to create and produce. Factors predisposing to traumatophilia are frequently present in children with high potential. Trauma may take the form of an internal or external reality, a relationship or indeed any form of stimulation that is experienced particularly intensely by these individuals. Cournut [12] stresses that “from the economic point of view, it matters little whether the excessive excitation is in the nature of terror or pleasure. What counts is its possible downgrading in the event of an economic breakdown of a regime of constancy.” This same narcissistic vulnerability can even compromise the individual's access to temporality and relationship with time, for the close connection between sexuality and libidinal cathexis on the one hand, and loss and even death on the other, means that time is processed differently. This lends a particular coloring to the knowledge drive, which becomes a veritable “exhumation drive” concerned with origins and absence. Thus, to quote Andre [33] high potential and fear of loss are bound together by “an overflowing, a traumatic libidinal effraction, rather than the reality of a loss.” As threatening representations are less thoroughly repressed in children with high intellectual potential, we can add a fourth possibility to the three outcomes of the knowledge drive described by Weismann-Arcache [28], whereby repression does not overcome the drive, and the infantile sexual theories, followed by the family theory, continue to operate on a low light, fueling the need to theorize, to historicize, and to think. Overdeveloped intelligence may not always be pathological, but it is an antitraumatic response to the dangers and losses that punctuate our lives. This, according to Freud [34, 35], herself the daughter of a creative explorer, “would explain the fact that instinctual danger makes human beings intelligent. In periods of calm in the instinctual life, when there is not danger, the individual can permit himself a certain degree of stupidity. In this respect, instinctual anxiety has the familiar effect of objective anxiety” [34, page 152].

## References

[1] Liratni M, Pry R (2011). Gifted children: psychopathology, socialization and adaptive behaviors. *Neuropsychiatrie de l’Enfance et de l’Adolescence*.

[2] Tordjman S, Lubart T (2006). Approche psychopathologique des enfants surdoués. *Enfants exceptionnels, Précocité intellectuelle, haut potentiel et talent*.

[3] Tordjman S (2010). *Aider les enfants à haut potentiel en difficulté: Repérer et comprendre, évaluer et prendre en charge*.

[4] Pedinielli JL, Bernoussi A (2008). *Les états dépressifs*.

[5] Anzieu D (1981). *Le corps de l’œuvre*.

[6] Weismann-Arcache C (2006). Hétérogénéité ou dysharmonie, approche clinique du fonctionnement mental des enfants à haut potentiel. *Bulletin de Psychologie*.

[7] Aristotle (2011). *L’homme de génie et la mélancolie*.

[8] Freud S (1997). Le créateur littéraire et la fantaisie. *L’inquiétante étrangeté et autres essais*.

[9] Bergeret J (1992). *La dépression et les états-limites*.

[10] Janin C (2005). Au cœur de la théorie psychanalytique: Le traumatisme. *Le traumatisme psychique, organisation et désorganisation, Monographies de la Revue Française de Psychanalyse*.

[11] Golse B, Baubet T, Lachal C, Ouss Ryngaert L, More MR (2000). Preface. *Bébés et traumas*.

[12] Cournut J (2002). *L’ordinaire de la passion: Névroses du trop, névroses du vide*.

[13] Bailly L, Baubet T, Lachal C, Ouss Ryngaert L, Moro MR (2006). Traumatisme psychique chez le jeune enfant et théories sociales infantiles. *Bébés et traumas*.

[14] Weismann-Arcache C (2009). *Les surdoués, du bébé à l’adolescent. Les destins de l’intelligence*.

[15] Weismann-Arcache C (2010). L’adolescent savant: Penser la mort pour rêver d’amour. *Adolescence*.

[16] Ferenczi S (1974). Le rêve du nourrisson savant. *Psychanalyse III*.

[17] Ferenczi S (1982). Confusion de langue entre les adultes et l’enfant. **Œ*uvres complètes*.

[18] Pirlot G Pensée/trauma/vide: De Pascal, Descartes, à la question de l’âme et la clinique du vide chez les sujets limites.

[19] Tordjman S, Nevoux G, Tordjman S (2010). Le dessin comme support d’étude des liens entre créativité et psychopathologie. *Le dessin des enfants à haut potentiel De la créativité à la psychopathologie*.

[20] Weismann-Arcache C Quand le génie s’oppose à l’esprit. L’intelligence comme limite à la relation et au transfert?. *Cliniques frontalières*.

[21] Denis P (2001). *Éloge de la bêtise*.

[22] Weismann-Arcache C (2011). L’intelligence "surdéveloppée", une héritière de la dépression infantile. *Psychologie Clinique et Projective*.

[23] Bergeret J (2000). *Psychologie pathologique théorique et clinique*.

[24] Chabert C, Chabert C (2009). Cliniques de la dépression. Métapsychologie de la perte. *Narcissisme et dépression Traité de psychopathologie adulte*.

[25] André J, Braconnier A, Golse B (2010). De la perte à la sexualité infantile. *Dépression du bébé, dépression de l’adolescent*.

[26] Brenot P (2007). *Le génie et la folie*.

[27] Green A (1980). La mère morte. *Narcissisme de vie, narcissisme de mort*.

[28] Weismann-Arcache C, Marty F (2007). Le Petit Hans était-il surdoué? Traumatisme, phobie et symbolisation. * Transformer la violence*.

[29] Freud S (2000). *Un souvenir d’enfance de Léonard de Vinci*.

[30] Anzieu D, Besdine M (1974). *Psychanalyse du génie créateur*.

[31] Lowenfeld H (1977). Traumatisme psychique et expérience créatrice chez l’artiste. *Psychanalyse à l’Université*.

[32] Emmanuelli M (1994). Incidence of narcissism in adolescent thought processes. *Psychiatrie de l’Enfant*.

[33] Andre J (1995). La perte d’amour. *Problématiques du féminin, Psychologie clinique et projective*.

[34] Freud A (1985). *Le moi et les mécanismes de défense*.

[35] Freud A (1995). *The ego and the mechanisms of defence*.

